# Pigment epithelium-derived factor (PEDF): a novel trophoblast-derived factor limiting feto-placental angiogenesis in late pregnancy

**DOI:** 10.1007/s10456-016-9513-x

**Published:** 2016-06-08

**Authors:** Jelena Loegl, Erika Nussbaumer, Ursula Hiden, Alejandro Majali-Martinez, Nassim Ghaffari-Tabrizi-Wizy, Silvija Cvitic, Ingrid Lang, Gernot Desoye, Berthold Huppertz

**Affiliations:** Department of Obstetrics and Gynecology, Medical University of Graz, Graz, Austria; Institute of Cell Biology, Histology and Embryology, Medical University of Graz, Graz, Austria; Institute of Pathophysiology and Immunology, Medical University of Graz, Graz, Austria

**Keywords:** Trophoblast, Endothelium, Angiogenesis, Placenta, PEDF, VEGF

## Abstract

The rapidly expanding feto-placental vasculature needs tight control by paracrine and endocrine mechanisms. Here, we focused on paracrine influence by trophoblast, the placental epithelium. We aimed to identify differences in regulation of feto-placental angiogenesis in early versus late pregnancy. To this end, the effect of conditioned media (CM) from early and late pregnancy human trophoblast was tested on network formation, migration and proliferation of human feto-placental endothelial cells. Only CM of late pregnancy trophoblast reduced network formation and migration. Screening of trophoblast transcriptome for anti-angiogenic candidates identified pigment epithelium-derived factor (PEDF) with higher expression and protein secretion in late pregnancy trophoblast. Addition of a PEDF-neutralizing antibody restored the anti-angiogenic effect of CM from late pregnancy trophoblast. Notably, human recombinant PEDF reduced network formation only in combination with VEGF. Also in the CAM assay, the combination of PEDF with VEGF reduced branching of vessels below control levels. Analysis of phosphorylation of ERK1/2 and FAK, two key players in VEGF-induced proliferation and migration, revealed that PEDF altered VEGF signaling, while PEDF alone did not affect phosphorylation of ERK1/2 and FAK. These data suggest that the trophoblast-derived anti-angiogenic molecule PEDF is involved in restricting growth and expansion of the feto-placental endothelium predominantly in late pregnancy and targets to modulate the intracellular effect of VEGF.

**Electronic supplementary material:**

The online version of this article (doi:10.1007/s10456-016-9513-x) contains supplementary material, which is available to authorized users.

## Introduction

During pregnancy the feto-placental vascular system has to adapt rapidly to the growing needs of the fetus, as inadequate feto-placental vascular development and angiogenesis result in pregnancy failure or pregnancy pathologies, i.e., fetal growth restriction or preeclampsia [1]. Placental vascular development and expansion are stimulated by growth promoting and pro-angiogenic factors. Such factors include vascular endothelial growth factor (VEGF), placental growth factor (PLGF) and angiopoietin 1 (ANG1) [2–4]. The angiogenic process, however, also requires control and is, therefore, balanced by anti-angiogenic proteins of which soluble Fms-related tyrosine kinase-1 (sFlt1) is most prominent [5, 6].

The outermost layer of placental villi is formed by the syncytiotrophoblast, which is generated by subjacent cytotrophoblast cells. The trophoblast is a source of soluble pro- and anti-angiogenic factors, suggesting a key role in regulating feto-placental vascular growth. A prominent example of a trophoblast-derived pro-angiogenic factor is VEGF, which has been suggested to promote growth of feto-placental vessels [7]. A natural endogenous VEGF inhibitor produced by the trophoblast is sFlt1. sFlt1 is a truncated splice variant of Flt1 (VEGF receptor 1), lacking the membrane-spanning and intracellular kinase domain [8, 9]. Intuitively, one would hypothesize that in early pregnancy, i.e., in the first trimester, feto-placental angiogenesis is highly stimulated as is placental growth and development, while in late pregnancy, i.e., in the third trimester, when placental growth decays, angiogenesis has to be progressively limited. Support for this hypothesis comes from strong VEGF expression in trophoblasts during early pregnancy and lower levels thereafter, demonstrating temporal changes in the growth regulation of feto-placental vessels [7].

Here we hypothesized that the trophoblast secretes anti-angiogenic factors, which increase in late pregnancy to limit angiogenesis. Therefore, we determined the paracrine effect of primary human trophoblast from early versus late pregnancy on the angiogenic potential of isolated feto-placental endothelial cells. Conditioned media of trophoblast from early and late pregnancy were added to endothelial cells isolated from human placenta to study key processes of angiogenesis. Among the proteins secreted by trophoblast in late pregnancy, pigment epithelium-derived factor (PEDF) was identified as a novel trophoblast-derived suppressor of feto-placental angiogenesis.

## Materials and methods

### Cell isolation, culture and characterization

Placentas from third trimester were obtained after uncomplicated vaginal delivery or cesarean section, between gestational weeks 36 and 40. First trimester placentas were obtained from elective pregnancy terminations, between gestational weeks 9 and 11. The study was approved by the local ethics committee, and all patients gave written informed consent (approval number 25-008 ex 12/13 and 12-095 ex 01/02).

#### Primary third trimester feto-placental endothelial cells

Primary placental endothelial cells were isolated from third trimester placentas (*n* = 14) following a standard protocol [10]. In brief, chorionic blood vessels were dissected and endothelial cells isolated by perfusion with a collagenase/dispase (Roche, Germany) solution. Cells were resuspended in endothelial basal medium (EBM, Lonza, USA) supplemented with the EGM-MV BulletKit (Lonza) containing gentamicin/amphotericin, hydrocortisone, human epidermal growth factor (EGF), bovine brain extract and fetal bovine serum (FBS) (Thermo Scientific, USA), and plated on culture plates precoated with 1 % gelatin (Sigma-Aldrich, USA). All cell preparations were subjected to immunocytochemical characterization for identity, purity and functionality. Isolated feto-placental endothelial cells were grown at 37 °C and 21 % oxygen and used up to passage 10.

#### Primary third trimester trophoblasts (TTB)

Primary villous trophoblasts were isolated from third trimester placenta (*n* = 6) as described [11, 12]. Briefly, minced villous tissue was digested with a trypsin/dispase/DNase solution (Gibco, UK; Roche, Germany; Sigma, USA). After Percoll gradient (Sigma) centrifugation a negative selection with MCA-81-conjugated magnetic beads (Sigma) was performed to obtain pure cultures of villous trophoblast. Isolated villous trophoblasts were cultured in DMEM (Gibco) supplemented with 10 % FBS, 20 mM HEPES pH 7.4 (Sigma) and penicillin/streptomycin (Sigma). After isolation cells were tested for viability and differentiation by measuring β-human chorionic gonadotropin secretion (Dade Behring, USA) [13]. Purity was determined by immunocytochemical staining for the trophoblast marker cytokeratin 7 (CK7, Dako, Denmark) (see Table 1 for details on antibodies) [11]. The cells were plated on plastic dishes and cultured at 37 °C and 21 % oxygen.

**Table 1:** Genes encoding anti-angiogenic molecules expressed in first and third trimester trophoblast (signal > 200) as determined by microarray analysis

Gene symbol	Gene name	FTB	TTB
ANGPT2	Angiopoietin 2	735	a
ARRB1	Arrestin beta 1	336	446
CD59	CD59 complement fragment	713	724
COL4A1	Collagen type IV alpha 1	9985	2830
COL4A2	Collagen type IV alpha 2	7011	2570
COL4A3	Collagen type IV alpha 3/tumstatin	a	a
COL18A1	Collagen type XVIII alpha 1	a	a
CXCL2	Gro-beta	307	377
CXCL10	Chemokine ligand 10	227	a
FN1	Fibronectin	1165	681
FBLN5	Fibulin5	a	a
HSPGBM	Heparan sulfate proteoglycan of basement membrane	744	a
IFNA1	Interferon alpha	a	a
IFNB1	Interferon beta	a	a
IFNG1	Interferon gamma	a	a
IL4	Interleukin-4	a	a
IL12A	Interleukin-12A	a	a
IL12B	Interleukin-12B	a	a
IL18	Interleukin-18	a	a
KISS1	Kisspeptin	1121	a
LECT1	Leukocyte cell-derived chemotaxin (chondromodulin)	a	a
NRP1	Neuropilin 1	a	a
PF4	Platelet factor 4	a	a
PLG	Plasminogen fragment	a	a
PRL	Prolactin	a	233
RNH	Placental ribonuclease inhibitor	1932	2377
SDC3	Syndecan 3	212	a
SERPINB5	Maspin	a	a
SERPINC1	Serpin peptidase inhibitor, clade C (anti-thrombin III)	a	a
SERPINE1	Plasminogen activator inhibitor/serine peptidase inhibitor	11,887	9413
SERPINF1	Pigment epithelium-derived factor (PEDF)	776	2914
SPARC	Secreted protein, acidic, cysteine rich	2458	555
SPP1	Osteopontin	228	323
TGFB1	Transforming growth factor beta	a	a
THBS1	Thrombospondin 1	a	a
THBS2	Thrombospondin 2	a	a
TIMP1	Metalloproteinase inhibitor 1	1226	292
TIMP2	Metalloproteinase inhibitor 2	2849	2711
TIMP3	Metalloproteinase inhibitor 3	11,176	7901
TIMP4	Metalloproteinase inhibitor 4	a	a
TNNI3	Troponin I	a	a
TNFSF15	Tumor necrosis factor ligand superfamily, member 15/VEGI	a	a

*a* absent

#### Primary first trimester trophoblast cells (FTB)

First trimester villous trophoblasts were isolated (*n* = 4) by enzymatic digestion with trypsin/dispase. Percoll centrifugation and negative magnetic bead immunopurification with the anti-leukocyte marker CD45 (Invitrogen, Norway) and anti-fibroblast marker CD90 (Dianova, Germany) were performed as described earlier [14]. Purity was checked by immunocytochemical staining for cytokeratin 7 (CK7, Dako). After isolation first trimester trophoblasts were resuspended in keratinocyte medium (Gibco) supplemented with the keratinocyte SFM kit (Gibco) containing epidermal growth factor (EGF1-35), bovine pituitary extract (BPE) and FBS. Cells were seeded on plastic dishes and cultured at 37 °C and 21 % oxygen.

### Conditioned medium

Freshly isolated first and third trimester trophoblasts were seeded at a density of 3 × 10^6^ cells per 2 ml in their appropriate medium to recover. After 24 h the medium was changed to DMEM/EBM (DE, 1:1) with 7.5 % FBS. After another 48-h incubation, the conditioned medium (CM) was aspirated and centrifuged for 5 min with 300*g* to remove dead cells and cell debris. CM was aliquoted and stored at −80 °C. CM was pooled to enable comparable testing with various assays using the same CM pool. At least two pools of first and third trimester trophoblast from two to four different isolations were used. As a control (non-CM), DMEM/EBM with 7.5 % FBS was incubated at the same conditions.

### In vitro network formation assay

To observe network formation, 1 × 10^4^ feto-placental endothelial cells were resuspended in conditioned/treatment medium and plated on growth factor-reduced Matrigel (BD Bioscience, USA). Tube-like structures were visualized after 12-h incubation by a Zeiss Cell Observer microscope with an AxioCam HRm camera and an A-Plan 5x/0.12 Ph0 objective using the software AxioVision (Carl Zeiss Imaging Solutions GmbH). For quantification the total tube length, the branching points and the number of meshes were analyzed by the ImageJ software (NIH) using the AngioJ-Matrigel assay plugin, kindly provided by Diego Guidolin (Department of Human Anatomy and Physiology, Section of Anatomy, University of Padova, Italy) [15]. Thereby, total network length, number of branching points and meshes were counted. As representative parameter total tube length can be used because branching points and number of meshes show the same trend.

### Migration/chemoattraction assay

Migration/chemoattraction of medium was observed using a 96-well chemotaxis microplate system (Neuro Probe Inc, UK). After serum starvation for 3 h in EBM, 1 × 10^4^ cells per well were placed in the upper part of the chemotaxis system, which was separated from the lower well by a fibronectin-coated polycarbonate filter with 8-µm pores. Cells were allowed to migrate toward chemoattractants in the lower well (CM) for 4 h at 37 °C. As positive control, DE medium supplemented with FBS and growth factors (EGM-MV BulletKit, Lonza) was used. The upper surface of the filter was wiped clean of non-migrating cells. Cells were fixed with 4 % formaldehyde and stained with DAPI (Invitrogen, USA). Subsequently, the microplate was observed by a Zeiss Axioplan fluorescence microscope and a 10× objective using the AxioVision software (Carl Zeiss Imaging Solutions GmbH). From each filter well 35 pictures were taken. Out of these, 7 pictures were randomly selected and analyzed using DotCount v1.2 (online provided by Martin Reuter, MIT).

### Proliferation assay

Proliferation of feto-placental endothelial cells was assessed using the BrdU ELISA kit (Cyclex, Japan) according to the manufacturer’s recommendations. 6 × 10^3^ cells per well were seeded in a 96-well plate. After 24 h, the medium was changed to the conditioned/treatment medium and cells were incubated for another 24 h. Subsequently, BrdU was added to a final concentration of 10 µM and incubated for 2 h. Cells were fixed, denaturized and incubated with the monoclonal antibody against BrdU. Absorbance was measured immediately at 450/540 nm using the FluoSTAR Optima 413 spectrofluorometer (BMG Lab technologies, Germany).

### LDH assay

Cytotoxicity of conditioned/treatment medium on feto-placental endothelial cells was tested by measurement of released lactate dehydrogenase (LDH, Takara, Japan) according to the manufacturer’s instructions. 6 × 10^3^ cells per well were seeded in a 96-well plate with the conditioned/treatment medium for 24 h. Absorbance was measured immediately at 490/650 nm using the Spectromax 250 molecular devices microplate reader (MWG-Biotech, Germany).

### Chick chorioallantoic membrane (CAM) assay

To determine the effect of CM on angiogenesis, the ex ovo chorioallantoic membrane (CAM) assay was performed. Briefly, fertilized white leghorn chicken (*Gallus domesticus* L.) eggs (Schropper GmbH, Gloggnitz, Austria) were incubated for 3 days at 37.6 °C and 70–75 % relative humidity (J. Hemel Brutgeräte, Am Buschbach, Germany). Eggs were then opened into plastic weigh boats covered with square Petri dishes and returned to the incubator. On day ten, six on-plants were placed on the CAM vasculature. The on-plants consisted of a silicone ring containing either FTB CM, TTB CM or non-conditioned control medium, each on four different eggs. On day 3, vascularization of the on-plants was scored by a blinded observer using a five partite scale between −2 and +2.

The anti-angiogenic potential of PEDF in combination with VEGF was evaluated. Silicone rings contained either collagen (1 mg/ml) mixed with PEDF (10 ng/ml) or VEGF (25 ng/ml), or both, in DMEM/EBM supplemented with 7.5 % FCS. As control, collagen mixed with medium alone was used. For both settings, vessel sprouting was monitored under a microscope (Olympus stereomicroscope SZX16, Tokyo, Japan) immediately after application of the silicone ring on day 0 and each 24 h for 4 days.

### RNA isolation, array hybridization and data analysis

RNA was isolated with TRIzol (MRC, Cincinnati, OH, USA) followed by quality assessment using a bioanalyzer (Agilent, Palo Alto, CA, USA). Experimental procedures and data analysis followed recommended standards [16]. Total RNA from ten preparations per cell type (feto-placental endothelial cells: GEO accession number GSE59126; early pregnancy trophoblast: GEO accession number GSE59126; late pregnancy trophoblast: GEO accession number GSE69086), isolated from different placentas, was pooled. Using 5 μg of pooled RNA, cDNA was synthesized (SuperScript Double-Stranded cDNA Synthesis Kit; Invitrogen, Carlsbad, CA, USA), transcribed in vitro (RNA Transcript Labeling Kit; Enzo diagnostics, Farmingdale, NY, USA) and then fragmented. To test the quality of the cRNA, it was hybridized against Test-3 arrays (Affymetrix, Santa Clara, CA, USA). As samples passed the quality criteria (bioC, bioD and cre were present, and the 3′:5′ ratio of the polyA controls was <3), the cRNAs were hybridized against Affymetrix HU133A chips. RNA preparation and hybridization followed the Affymetrix user manual. Data analysis of raw data was normalized globally and processed with Microarray Suite, version 5.0 (Affymetrix) and Data Mining Tool (Affymetrix) software [17]. Genes that met the following three criteria were classed as being differentially expressed: (1) fold change ≥1.5 or ≤−1.5; (2) change in *p* value ≥0.992 or ≤0.008; and (3) at least one signal intensity (control or treatment) >100. Annotations were obtained from NetAffx (available at http://www.affymetrix.com, last accessed in December 2013).

### Quantitative reverse transcription PCR (RT-qPCR)

Total RNA was isolated using the RNeasy Mini Kit (Qiagen, Hilden, Germany). The quality and integrity of the RNA was determined by the ratio of spectrophotometric absorbance 260 nm/280 nm measured with the Scandrop 250 (Analytik Jena AG, Germany). The cDNA was synthesized from 250 ng total RNA according to the manufacturer’s instructions (SuperScript II Reverse Transcriptase protocol from Invitrogen, USA). 10 ng/µl of cDNA were used on a total reaction volume of 10 µl in the ABI Prism 5700 Sequence Detection System. RT-qPCR for PEDF was performed using the TaqMan assay Hs01106937_m1 (Applied Biosystems, CA, USA). Mean expression of the housekeeping gene ribosomal protein L30 (RPL30 Hs00265497_m1; Applied Biosystems) was used to normalize gene expression with $2^{{-\Delta\Delta c_{\text{t}}}}$ method.

### Quantification of PEDF and sFlt1

The PEDF and sFlt1 concentrations in CM were measured using immunoassays (Biovendor R&D Products, Minneapolis, USA) according to the manufacturer’s instructions.

### Neutralizing anti-PEDF antibody

Trophoblast-released PEDF in CM was blocked by using a neutralizing anti-PEDF antibody (BioProducts, Middletown, USA). A non-specific antibody (BioRad) served as isotype control. Antibodies were used at a concentration of 5 µg/ml.

### Treatment with pro- and anti-angiogenic factors

For all treatments and dilutions DMEM/EBM with 7.5 % FBS was used. Pigment epithelium-derived factor (PEDF, Prospec, Israel) was used at final concentrations of 0.05; 0.25; 0.5; 2.5; 5; and 10 ng/ml. The concentrations of 5 and 10 ng/ml were also combined with 25 ng/ml VEGF (Sigma).

### Dot blot assay

To determine VEGF concentrations, total protein concentration of CM was determined by a BCA protein assay (Thermo Scientific), according to the manufacturer’s instructions. Hundred micrograms of each sample and increasing concentrations of VEGF (Sigma) were spotted on a nitrocellulose membrane and air-dried. For blocking of non-specific sites the membrane was soaked in 5 % non-fat milk for 30 min. Then, the membranes were incubated with anti-VEGF antibody (Proteintech, Manchester, UK, 1:1000) for 1 h and washed three times (10 min) and the secondary antibody (anti-rabbit, BioRad, California, USA, 1:1000) was applied for 30 min. After three washing steps, the membrane was incubated with SuperSignal West Femto Chemiluminescent Substrate (Thermo Scientific) for 5 min and observed by a BioRad lumino image analyzer. The signals were quantified by Alpha DigiDoc software and standards used to determine the concentrations of the samples.

### Immunofluorescence

For co-localization of soluble factors and their receptors to distinct cell types, 5-µm sections were cut and placed on Superfrost Plus slides (Menzel, Braunschweig, Germany). Paraffin-embedded sections were deparaffinized in xylene and rehydrated through a series of graded alcohol. Heat-induced antigen retrieval was performed in epitope retrieval solution at pH9 (Leica Biosystems Newcastle Ltd., Newcastle, UK). Slides were boiled in a pressure cooker for 7 min at 120 °C and allowed to cool down for 20 min before being rinsed in wash buffer [phosphate-buffered saline, 0.05 % Tween-20 (PBS/T), pH 7.4]. All further steps were performed at room temperature. Slides were incubated with an Ultra V-Block (Thermo Scientific) for 7 min. Then, slides were rinsed in PBS/T three times before applying both primary antibodies, i.e., the PEDF (BioProducts MD, Middletown, USA; 1 µg/ml), VEGF (Proteintech, 0.7 µg/ml) and VEGFR2 (Santa Cruz Biotechnology, USA; 0.2 µg/ml) in combination with the endothelial cell marker CD31 (Abcam, Cambridge, UK; 5 µg/ml) or the trophoblast marker CK7 (Thermo Scientific, Rockford, USA; 0.1 µg/ml), for 45 min. Primary antibodies were diluted in antibody diluent with background reducing components (Dako). Negative controls were incubated with non-specific IgG fractions of the appropriate isotype from mouse (IgG/IgM) (Dako) or with a negative control for rabbit IgG (Thermo Scientific). All incubation steps were performed in a dark, humidified chamber. After three washings in PBS/T slides were incubated for 30 min with fluorescent-labeled secondary antibodies (Alexa Fluor^®^ 555 goat anti-mouse IgG and Alexa Fluor^®^ 488 goat anti-rabbit IgG; both diluted 1:2000, Invitrogen, Lofer, Austria). Afterward, slides were stained with 4,6-diamidino-2-phenylindoledihydrochloride (DAPI; diluted 1:2000 in PBS; Invitrogen) for 10 min. Slides were rinsed in deionized water, air-dried and mounted with ProLong Gold anti-fade reagent (Invitrogen). Sections were assessed with a Leica DM 6000B microscope and photographed using an Olympus DP 72 Camera (Leica Microsystems, Wetzlar, Germany).

### Immunoblot analysis for VEGF and PEDF signaling

Feto-placental endothelial cells (150,000/well) were seeded in supplemented EBM gelatin-coated six-well plates. After 48 h, cells were serum starved for 4 h. Then, PEDF (10 ng/ml), VEGF (25 ng/ml) and the combination of both were added for 10 min. The VEGFR2 inhibitor Ki8751 (Calbiochem, Merck Millipore, Darmstadt, Germany) was added to a final concentration of 10 ng/ml 30 min prior to the VEGF treatment. Protein was isolated using RIPA buffer with complete protease inhibitor (Sigma, 1 tablet/10 ml) and used for immunoblot analysis for P-ERK1/2 (Tyr 576) (Millipore; 1:1000) and P-FAK (Tyr 397) (Cell Signalling, Merck Millipore; 1:1000). Antibodies against unphosphorylated ERK1/2 (Abcam; 1:1000) and FAK (Abcam; 1:1000) were used as loading controls.

### Statistical analysis

Data are expressed as mean ± SEM. Statistical analysis used SigmaPlot 12.0 software, and a *p* value of <0.05 was considered as significant. Statistical differences were assessed by Student’s *t* test (Shapiro–Wilk test for normal distribution; Mann–Whitney *U* test for nonparametric values) and ANOVA.

## Results

### Paracrine regulation of feto-placental endothelial cell angiogenesis by trophoblast from early versus late pregnancy

**Fig. 1:**
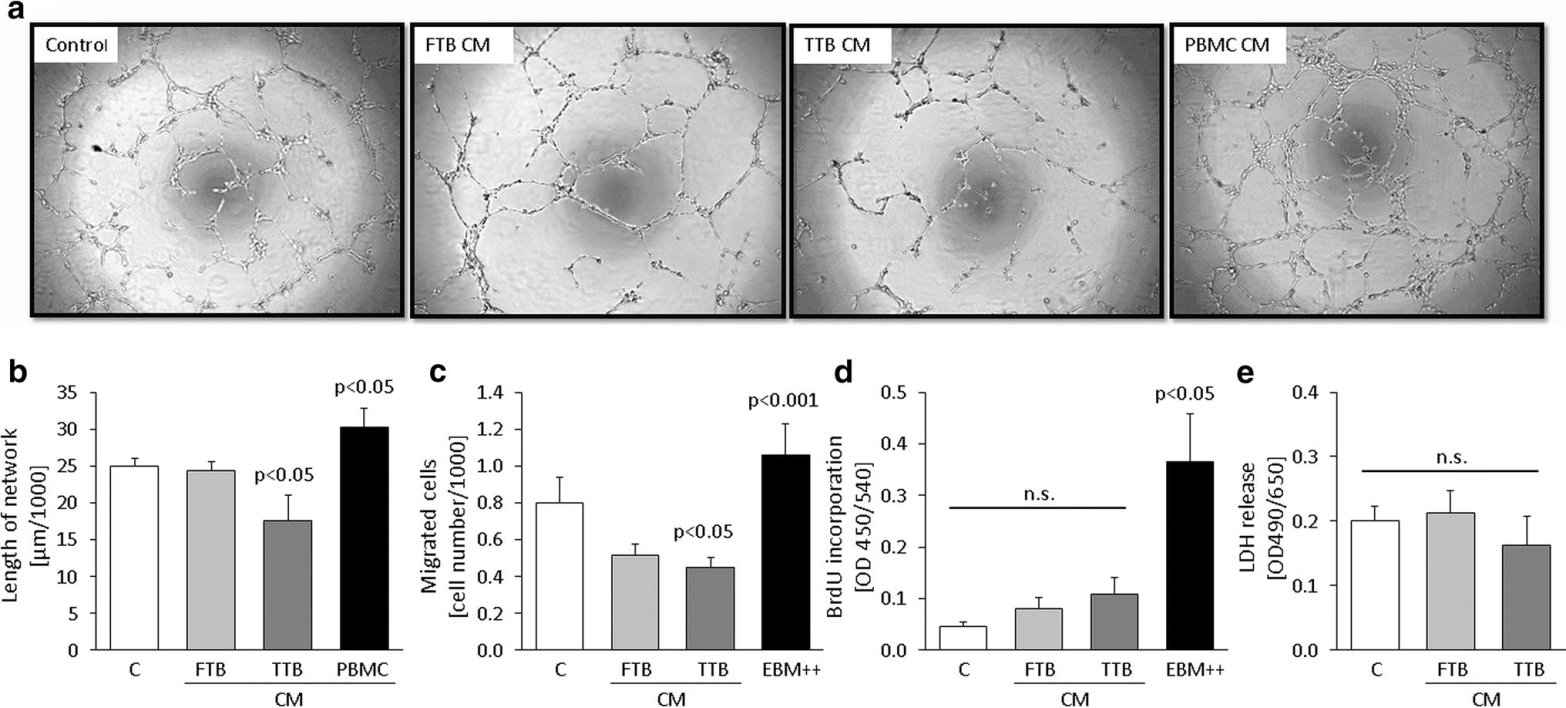
Effect of first (FTB) and third (TTB) trimester trophoblast-conditioned medium (CM) on network formation, migration, proliferation and survival of feto-placental endothelial cells. **a** Representative phase contrast light microscopic images show network formation of feto-placental endothelial cells after 12 h on growth factor-reduced Matrigel. CM of peripheral blood monocyte cells (PBMC) used as positive control. **b** Quantitative analysis of networks depicts the inhibitory effect of TTB CM on feto-placental endothelial cells (*n* = 6). **c** CM of FTB and TTB reduced migration of feto-placental endothelial cells compared to control (*n* = 6). Endothelial basal medium (EBM) supplemented with FBS and growth factors (EBM++) was used as positive control. **d** Proliferation was unchanged between early and late trophoblast CM (*n* = 4). EBM supplemented with FCS and growth factors (EBM++) was used as positive control. **e** FTB and TTB CM did not increase LDH release of feto-placental endothelial cells (*n* = 4). Data are given as mean ± SEM. Statistical analysis used the mean of the triplicate of the number of individual biological replicates. Control medium (*C*) = DMEM/EBM + 7.5 % FCS for 48 h at 37 °C without cells

To investigate the paracrine effect of trophoblast from early versus late pregnancy on angiogenesis, the influence of CM on network formation, migration, proliferation and survival of feto-placental endothelial cells was analyzed (Fig. 1). CM of trophoblast from late pregnancy (term trophoblasts; TTB) reduced network formation of feto-placental endothelial cells by 30 ± 14 % (*p* = 0.024) (Fig. 1a, b), while CM of trophoblast from early pregnancy (first trimester trophoblasts; FTB) had no effect. CM of peripheral blood monocyte cells (PBMC) used as positive control [18] stimulated network formation by 21 ± 10 % (*p* = 0.049). Similar results were obtained with CM that had been ultracentrifuged prior to use (not shown), indicating that soluble factors secreted by trophoblast induce the anti-angiogenic effects.

To determine paracrine regulation of trophoblast CM from early versus late pregnancy on migration, transwell migration assays were performed. TTB CM reduced migration by 44 ± 7 % (*p* = 0.026), while FTB CM reduced migration only by trend (Fig. 1c). Medium supplemented with FBS and growth factors was used as positive control and enhanced feto-placental endothelial cell migration by 32 ± 22 % (*p* = 0.007).

The effect of trophoblast CM from early and late pregnancy was further determined on proliferation, which is a further step of angiogenesis. CM of both, FTB and TTB, tended to increase BrdU incorporation as compared to control medium, but without reaching significance (Fig. 1d). Medium supplemented with FBS and growth factors was used as positive control and stimulated proliferation eightfold (*p* = 0.039).

Neither FTB nor TTB CM increased LDH release from feto-placental endothelial cells (Fig. 1e), indicating that the negative effect of CM on network formation and migration did not result from reduced cell survival and viability.

**Fig. 2:**
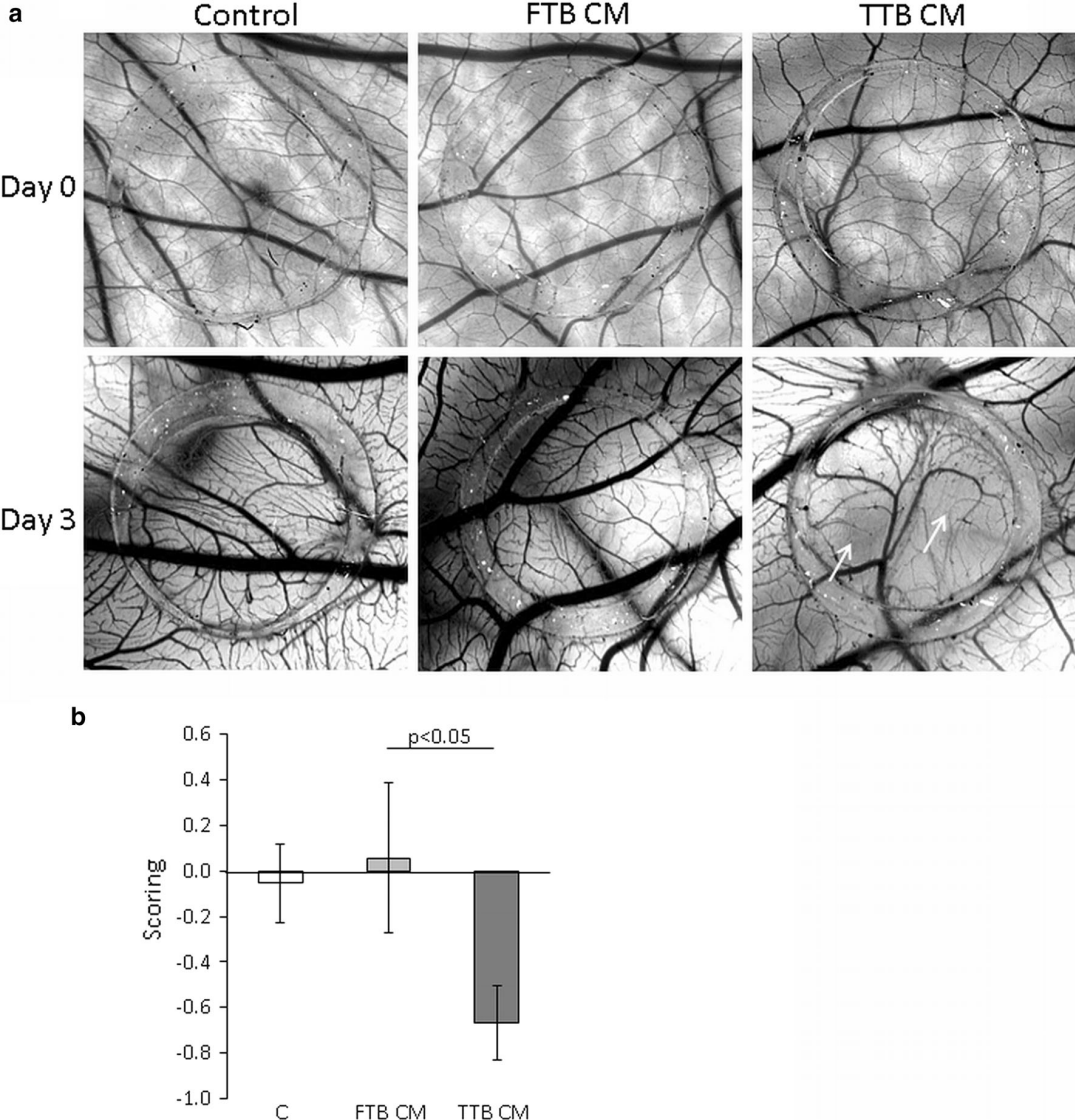
Anti-angiogenic effect of first (FTB) and third (TTB) trimester trophoblast-conditioned medium (CM) on the vessel formation of the chicken chorioallantoic membrane (CAM). CAMs were treated with on-plants (*silicone rings*) containing FTB CM, TTB CM or control medium. TTB CM reduced the tertiary and quaternary vessels (*white arrows*). Per condition, four different eggs were treated, each performed with six on-plants. **a** Representative pictures of each condition immediately after application of the silicone ring on day 0 and at day 3 of the treatments are shown. **b** Data were scored on day 3 of the treatment

In order to evaluate the effect of the CM in a physiological 3D model of embryonic angiogenesis, we employed chicken chorioallantoic membrane (CAM) assays. In parallel with the data obtained from the feto-placental endothelial cells, TTB CM reduced vasculogenesis (Fig. 2a, b).

### Anti-angiogenic molecules expressed by trophoblasts from early versus late pregnancy

**Fig. 3:**
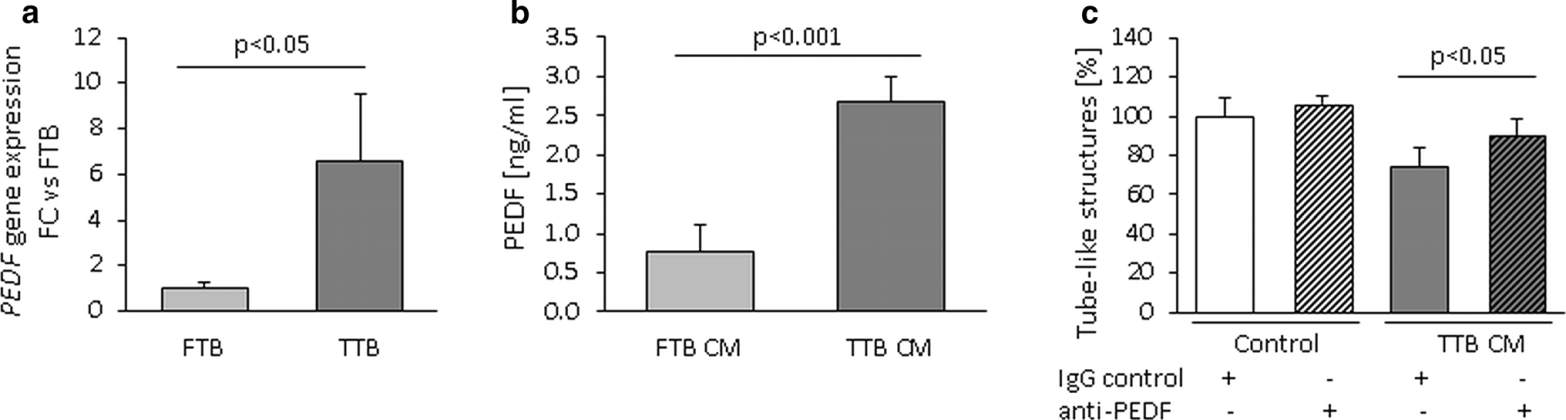
Expression and secretion of PEDF from first (FTB) and third (TTB) trimester trophoblasts. **a** Quantification of *PEDF* mRNA in FTB and TTB by real-time qPCR using the expression of the ribosomal protein RPL30 as internal control. **b** Quantification of PEDF in FTB versus TTB CM of measured with ELISA. **c** PEDF-neutralizing antibody significantly reduced the inhibitory effect of TTB CM on network formation of feto-placental endothelial cells. Data are given as mean ± SEM. Statistical analysis used the mean of the triplicate of the number of individual biological replicates. *n* (real-time qPCR) = 5, *n* (ELISA) = 8, *n* (network formation) = 3. Control medium (*C*) = DMEM/EBM + 7.5 % FCS for 48 h at 37 °C without cells

The reduction of network formation and migration in the presence of CM from late pregnancy indicates the presence of anti-angiogenic factors secreted by trophoblast. To identify potential targets causing the anti-angiogenic effect of late pregnancy CM, the expression of genes encoding angiogenesis-limiting factors was compared in FTB versus TTB. Thus, their transcriptomes were screened for 42 candidate angiogenesis-related genes obtained from the literature (Table 1) [19–42]. Of these, 21 were expressed either in early or in late pregnancy trophoblast, or both (as threshold for expression, a signal >200 was used). In general, the expression of anti-angiogenic factors was higher in early compared to late pregnancy trophoblast. Eight genes differed in their expression by less than twofold between early and late pregnancy trophoblast (Table 2) with only PEDF (pigment epithelium-derived factor) showing higher (3.8-fold) expression in late pregnancy trophoblast. Real-time qPCR confirmed the microarray results and revealed a fold change of 6.6 ± 2.9 (Fig. 3a). This suggests PEDF as a potential contributing factor to the anti-angiogenic paracrine effect of late pregnancy trophoblast, and the effect of PEDF on feto-placental endothelial cells was further studied.

**Table 2:** Genes encoding anti-angiogenic proteins differentially expressed between first or third trimester trophoblast (signal > 200, fold change >2 or <−2) as determined by microarray analysis

Gene symbol	Gene name	Fold change
SERPINF1/PEDF	Pigment epithelium-derived factor	−3.8
THBS1	Thrombospondin 1	2.6
TIMP1	Tissue inhibitor of metalloproteinases 1	4.2
SPARC	Secreted protein, acidic, cysteine rich	10.2
SDC3	Syndecan 3	11.8
TGFB1	Transforming growth factor beta 1	16.8
ANGPT2	Angiopoietin 2	17.5
KISS1	Kisspeptin	101.9

Positive fold change indicates higher expression in first trimester trophoblast, while a negative fold change indicates higher expression in third trimester trophoblast

*a* absent

### Identification of trophoblast-derived PEDF as negative regulator of network formation and proliferation in feto-placental endothelial cells

In line with mRNA expression, TTB secreted more PEDF (2.6 ng/ml/1 × 10^6^ cells) than the same number of trophoblasts from early pregnancy (0.9 ng/ml/1 × 10^6^ cells; *p* = 0.025) (Fig. 3b). This difference in PEDF remained similar when PEDF levels were normalized to total protein content of the CM (2.9-fold higher levels in TTB; *p* < 0.001).

To determine whether PEDF accounts for the anti-angiogenic effect of CM from late pregnancy trophoblast, in vitro network formation assays with late pregnancy trophoblast CM were repeated in the presence of PEDF-neutralizing antibody (Fig. 3c). TTB CM with unspecific IgG reduced in vitro network formation by 26 ± 10 % (*p* = 0.025). This reduction diminished in the presence of PEDF-neutralizing antibodies.

### Effect of PEDF on VEGF-activated network formation and proliferation of feto-placental endothelial cells

**Fig. 4:**
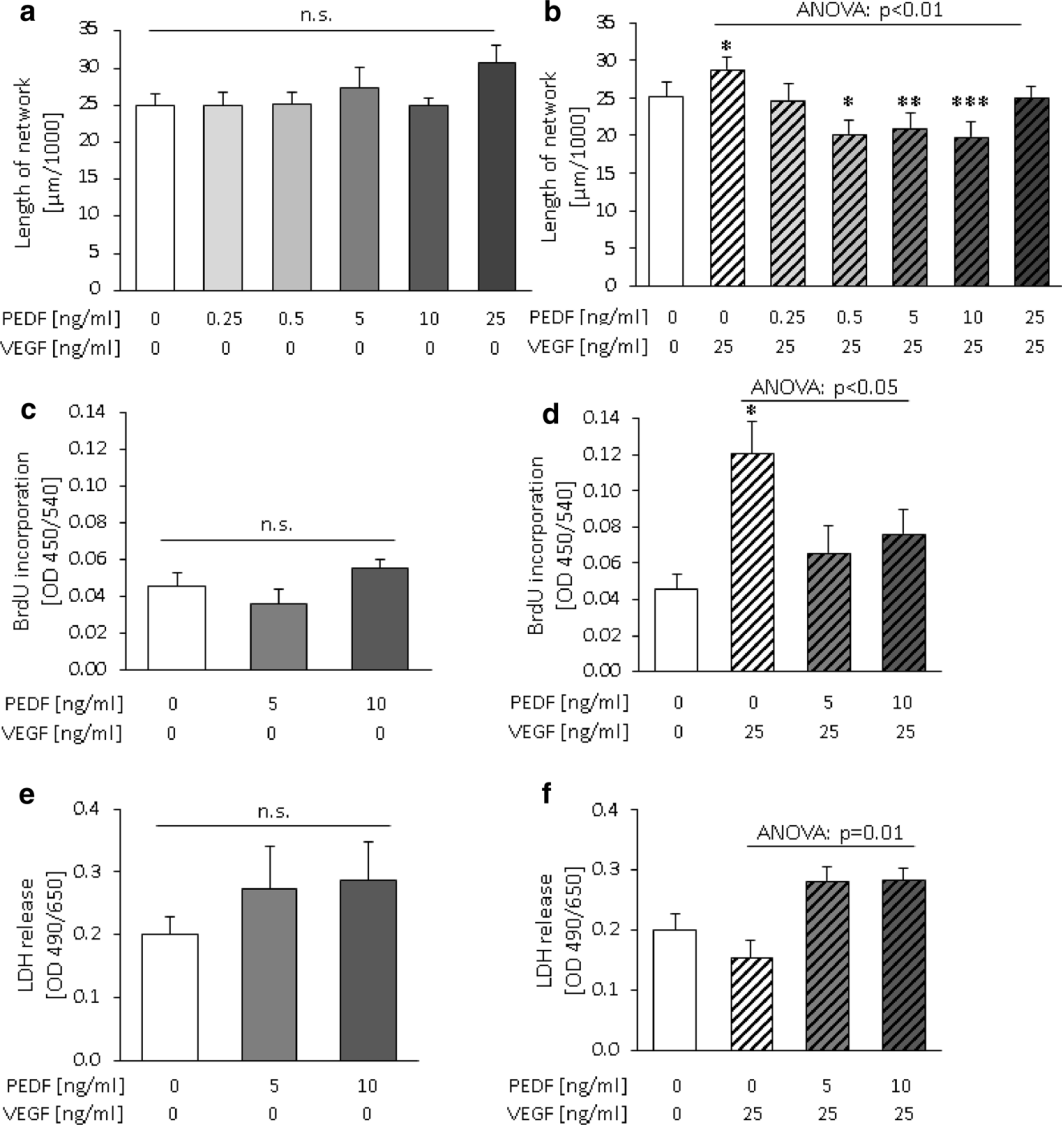
Effects of human recombinant PEDF on network formation, proliferation and survival of feto-placental endothelial cells depend on VEGF. **a**, **c**, **e** Addition of PEDF alone did not affect network formation, proliferation and LDH release of feto-placental endothelial cells. **b** In cells stimulated with VEGF (25 ng/ml), PEDF reduced network formation in a concentration-dependent manner, **d** reduced proliferation and **f** increased cell death. Data are given as mean ± SEM. Statistical analysis used the mean of the triplicate of the number of individual biological replicates. *n* (network formation) = 8, *n* (BrdU incorporation) = 9, *n* (LDH release) = 6. *n.s.* not significant; ***p < 0.001; **p < 0.01; *p < 0.05 compared to untreated control without VEGF

Stimulation of feto-placental endothelial cells with different concentrations (0.25–25 ng/ml) of human recombinant PEDF did not affect network formation (Fig. 4a). However, when 25 ng/ml VEGF was used to activate feto-placental endothelial cells for angiogenesis, PEDF reduced the VEGF effect in a dose-dependent manner to a maximum of 31 ± 7 % (Fig. 4b) when compared to VEGF alone. PEDF concentrations between 0.5 and 10 ng/ml reduced network formation to levels even below control levels, i.e., endothelial cells without any treatment. In the setting of these in vitro experiments with superphysiological VEGF levels and absence of other trophoblast-secreted angiogenesis-regulating factors, also PEDF levels similar to that in FTB CM reduced 2D network formation. Since 5 and 10 ng/ml PEDF produced the strongest and most significant effects, these concentrations were used for further experiments.

Also, proliferation and LDH secretion were not affected by PEDF treatment alone (Fig. 4c, e). However, when cells were co-stimulated with VEGF, PEDF (10 ng/ml) reduced the stimulatory effect of VEGF on proliferation (Fig. 4d) by 27 ± 8 % (*p* < 0.05). Moreover, PEDF increased LDH release by 42 ± 11 % (*p* < 0.001) (Fig. 4f) when compared to VEGF treatment alone.

In order to compare and relate the anti-angiogenic effects of PEDF with sFlt1, experiments were also performed with sFlt1 (Suppl. Fig. 1). As a molecule that sequesters VEGF, sFlt1 blocked the VEGF effects to control levels, but did not have further inhibiting effects.

**Fig. 5:**
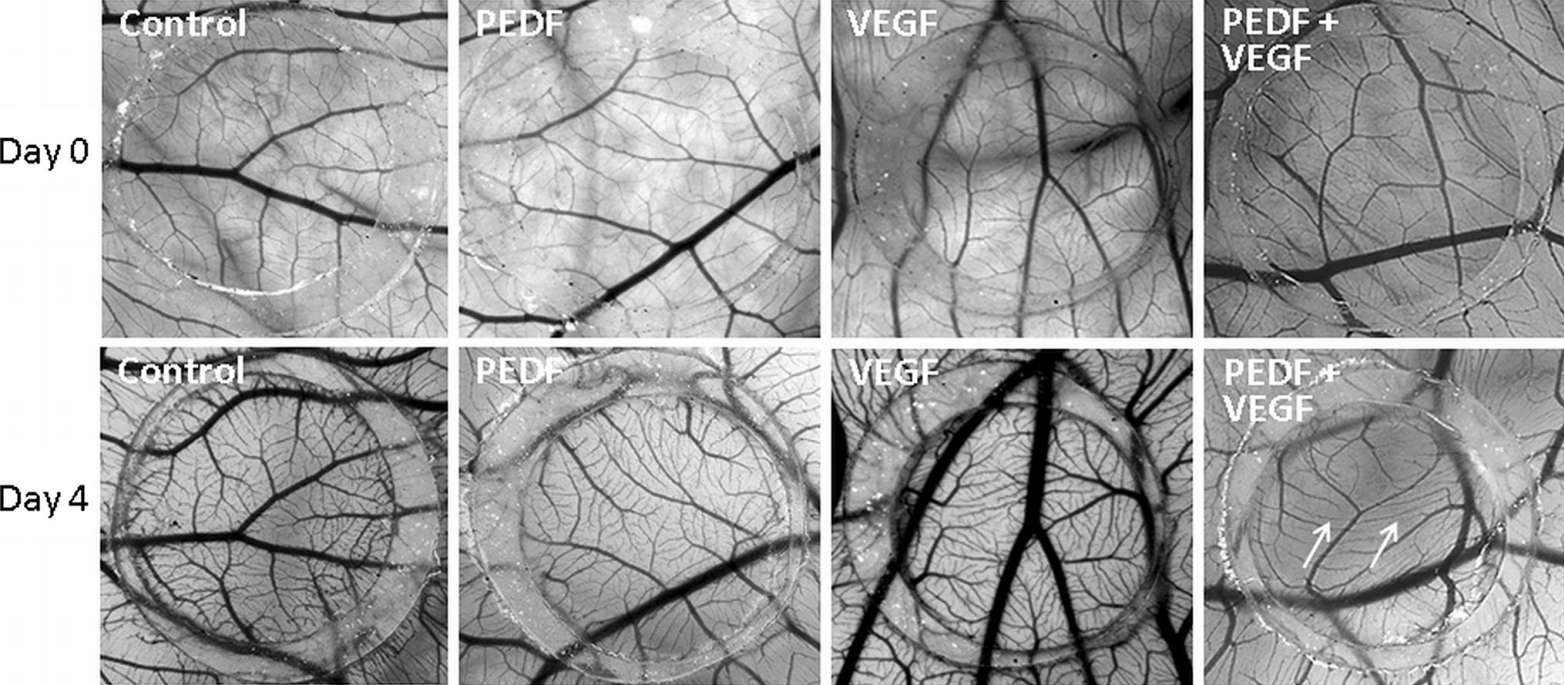
Anti-angiogenic activity of the combination of PEDF and VEGF on vessel formation of the chorioallantoic membrane (CAM). The same CAMs were treated with on-plants (*silicone rings*) containing 10 ng/ml PEDF with 25 ng/ml VEGF and control on-plants. Representative pictures of each condition immediately after application of the silicone ring on day 0 and at day 4 of the treatments are shown. After 4 days the vessel structure shows tertiary and quaternary vessels (*white arrows*). Addition of PEDF or VEGF alone did not affect vessel formation. Treatment with the combination of PEDF and VEGF CAM showed decreased angiogenesis by formation of fewer tertiary and quaternary vessels (*white arrows*)

The combined effect of PEDF and VEGF was also tested in CAM assays. Similar to the 2D network formation, the combined application of PEDF and VEGF reduced formation of tertiary and quaternary vessels after 4 days of treatment (Fig. 5), while PEDF and VEGF alone had no effect.

**Fig. 6:**
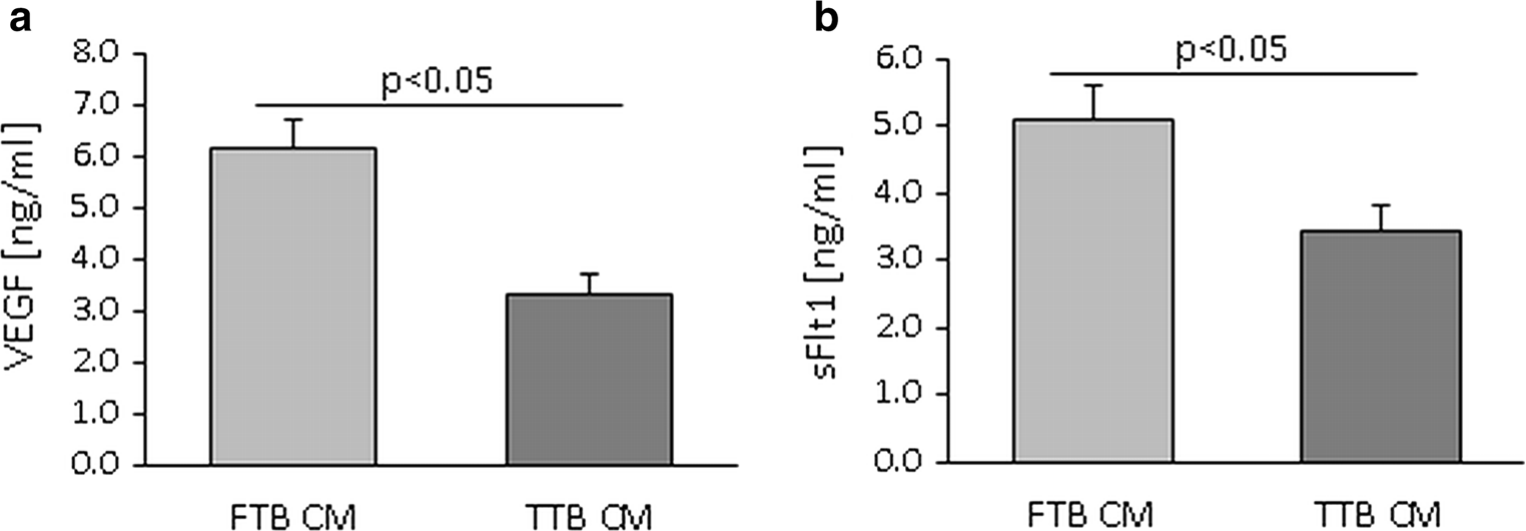
Protein secretion of VEGF and sFlt1 from first (FTB) and third (TTB) trimester trophoblast. **a** Quantification of VEGF in CM of FTB versus TTB measured by dot blot analysis. **b** Quantification of sFlt1 in CM of FTB versus TTB measured by ELISA. Data are given as mean ± SEM. Statistical analysis used the mean of the triplicate of the individual biological replicates. *n* (dot blot) = 4, *n* (ELISA) = 8

Since PEDF effect was dependent on the presence of VEGF, we measured VEGF levels and the levels of the VEGF capture molecule sFlt1 in FTB versus TTB CM. FTB secreted more sFlt1 (4.6 ng/ml/1 × 10^6^ cells) and VEGF (6.2 ng/ml/1 × 10^6^ cells) than TTB (sFlt1: 2.7 ng/ml/1 × 10^6^ cells, *p* = 0.028; VEGF: 3.3 ng/ml/1 × 10^6^ cells, *p* = 0.024) (Fig. 6a, b). The difference in VEGF and sFlt1 concentration remained when normalized to the total protein content of the CM (both factors had 1.6-fold higher levels in FTB CM; *p* = 0.02).

### Localization of PEDF, VEGF and VEGFR2 in placenta in late pregnancy

**Fig. 7:**
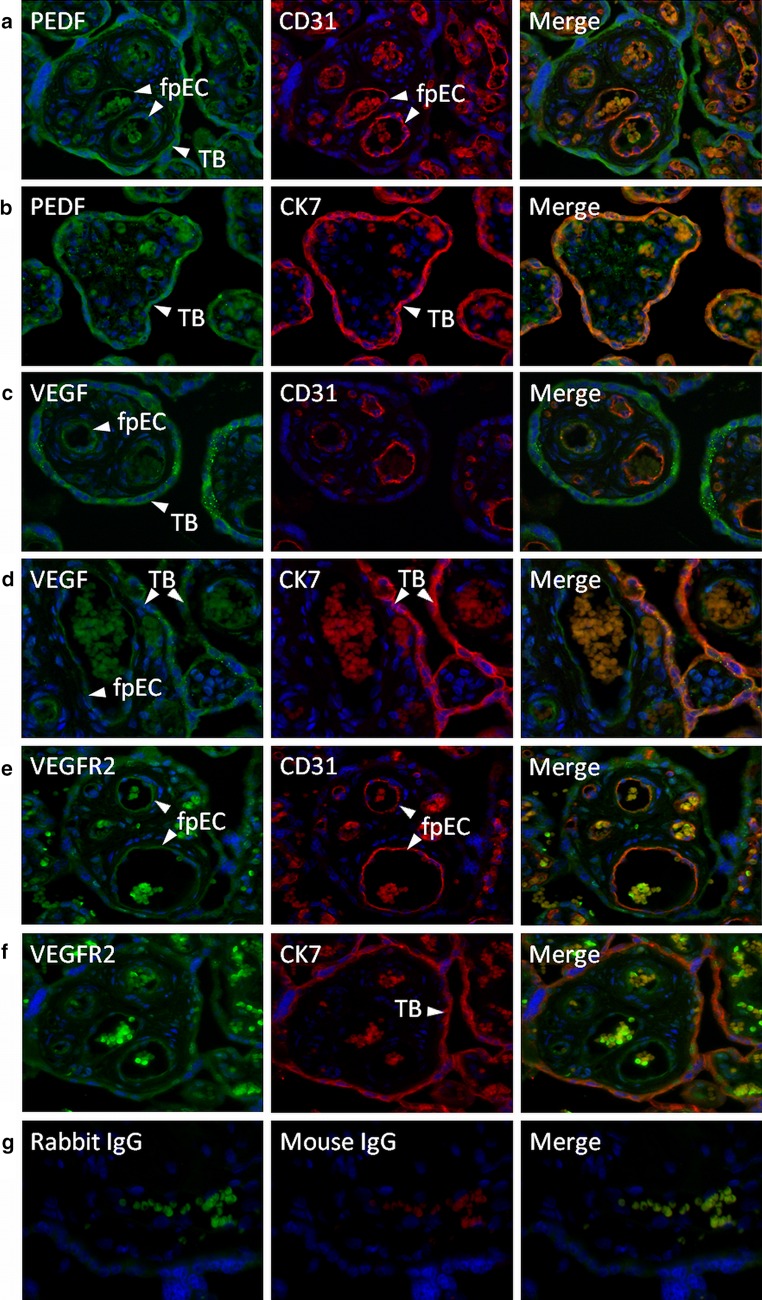
Protein expression of PEDF, VEGF and VEGFR2 in third trimester placenta. **a**, **b** PEDF and its receptor are predominantly expressed by villous trophoblast. **c**, **d** VEGF is expressed in trophoblasts and endothelial cells, similar to its pro-angiogenic receptor VEGFR2. **e** Isotype controls. Green staining: PEDF, VEGF, VEGFR2; red staining: CK7, CD31; blue staining: Dapi. TB = trophoblast layer, fpEC = feto-placental endothelial cells. Original magnification: ×200

Although PEDF mRNA expression in placenta was already demonstrated [43], the specific cell types expressing PEDF have not yet been identified. In line with the finding that isolated trophoblasts secrete PEDF, staining for PEDF revealed a prominent signal in the trophoblast, which was co-stained with the trophoblast marker cytokeratin 7 (Fig. 7b). Feto-placental endothelial cells co-stained with the endothelial cell marker CD31, and some other stromal cells showed a weak signal (Fig. 7a).

Several putative receptors for PEDF are known: adipose triglyceride lipase (ATGL) [44, 45], laminin receptor-1 (LR1) [46], beta subunit of F1-ATPase (ATP5B) [45] and low-density lipoprotein receptor-related protein 6 (LRP6) [47]. Gene expression analysis for the expression levels of these receptors in primary feto-placental endothelial cells revealed that all of these receptors are expressed (Table 3). Furthermore, PEDF can reduce VEGF-induced angiogenesis by competing with VEGF binding to the VEGF receptor 2 (VEGFR2; KDR) [48]. As the observed PEDF effects were dependent on the presence of VEGF, we also stained for VEGF and the VEGFR2. In line with published literature [49, 50], both VEGF and VEGFR2 were produced in the trophoblast and in feto-placental endothelial cells (Fig. 7c–f).

**Table 3:** Expression of genes encoding PEDF-binding partners in feto-placental endothelial cells as determined by microarray analysis

Gene symbol	Gene name	Signal
ATGL/PNPLA2	Adipose triglyceride lipase	2460
ATP5B	F_1_-ATP synthase beta subunit	37,640
KDR/VEGFR2	Kinase insert domain receptor	11,261
LR1/RPSA	Laminin receptor 1	62,601
LRP6	Low-density lipoprotein receptor-related protein 6	454

### PEDF modifies VEGF signaling in feto-placental endothelial cells

**Fig. 8:**
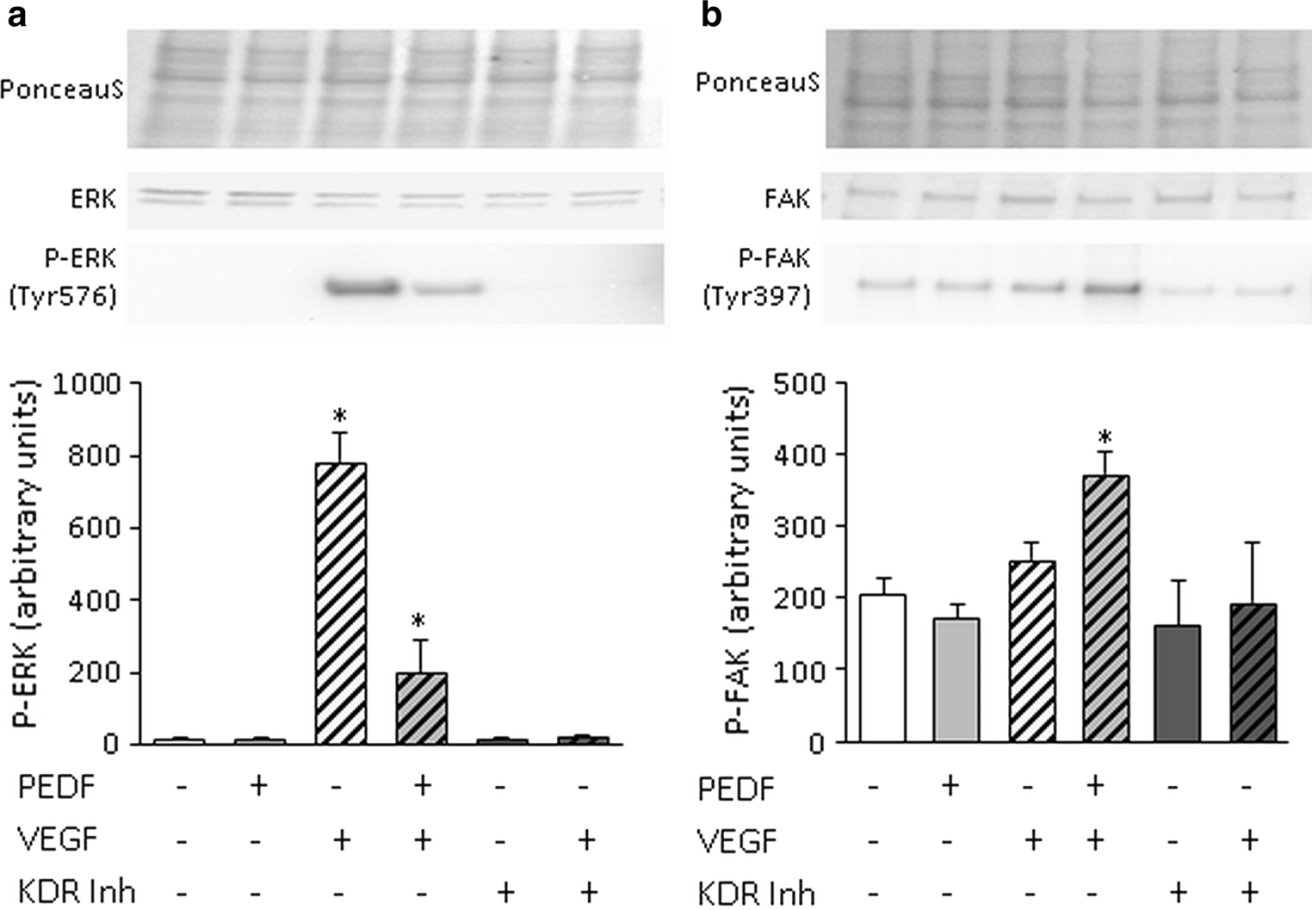
Signaling induced by the combination of PEDF and VEGF in feto-placental endothelial cells. Addition of PEDF (10 ng/ml) and VEGF (25 ng/ml) reduced VEGF-dependent phosphorylation of ERK1/2 at Tyr576 and caused phosphorylation of FAK at Tyr397 that was not activated by PEDF or VEGF alone. Addition of KDR inhibitor reduced VEGF effect to unstimulated levels. Signals were normalized to total blotted protein (PonceauS staining). Blots stained with antibodies against unphosphorylated forms of ERK1/2 and FAK were used as loading controls. Representative immunoblots are shown on *top*. Results are given as mean ± SEM of three different cell isolations, each in duplicates

Since PEDF exerts its anti-angiogenic effects on feto-placental endothelial cells only in combination with VEGF, we were interested in VEGF signaling in the presence and absence of PEDF. Therefore, we measured phosphorylation of two signaling molecules in response to either VEGF, PEDF or both. Two key molecules involved in proliferation and endothelial cell migration were chosen, i.e., extracellular signal-related kinase 1/2 (ERK1/2) [51] and focal adhesion kinase 1 (FAK) [52]. While PEDF inhibited the VEGF-induced phosphorylation of ERK1/2, FAK phosphorylation was not significantly stimulated by VEGF. However, in the presence of VEGF, PEDF increased FAK phosphorylation by 82 ± 14 % (Fig. 8).

## Discussion

This study identified PEDF secretion as a novel paracrine mechanism of human trophoblasts to limit feto-placental angiogenesis and vascular expansion in late pregnancy.

We report the following key findings: (1) CM from late pregnancy trophoblast reduced in vitro network formation and migration of primary feto-placental endothelial cells, and PEDF contributes to this effect. (2) The anti-angiogenic effect of PEDF on feto-placental endothelial cells depends on the concomitant activation of endothelial cells with VEGF where it modulates VEGF signaling.

Our study demonstrates the trophoblast as major source of PEDF in the placenta. PEDF is a non-inhibitory member of the serine protease inhibitor (SERPIN) gene family [43] with anti-angiogenic [53, 54], anti-tumorigenic [55] and anti-inflammatory [56] properties. Several binding partners were identified, through which PEDF exerts its anti-angiogenic effects: Adipose triglyceride lipase (ATGL) [44], low-density lipoprotein receptor-related protein 6 (LPR6) [47], F_1_-ATP synthase [45] and laminin receptor 1 (LR1) [46]. Furthermore, PEDF was shown to bind to VEGFR2 and to compete with VEGF binding [57, 58]. The dependence of PEDF effects on VEGF in our experiments highlights the prominent role of the VEGFR2 in anti-angiogenic PEDF effects and, thus, was further investigated.

Indeed, the finding that PEDF effect was depended on VEGF was observed also in other studies [57–61]. VEGF is part of the trophoblast secretome and acts pro-angiogenic when applied alone. PEDF not only blocked the stimulatory VEGF effect, but some PEDF doses reduced network formation even below control levels, i.e., endothelial cells without VEGF-stimulation. This indicates that PEDF can also act through mechanisms other than only interfering with VEGF action. This was supported by the finding that PEDF modulates VEGF signaling in different ways, i.e., by reducing VEGFR2-mediated ERK1/2 phosphorylation, and by increasing FAK phosphorylation, that was not affected by VEGF alone. The fact that PEDF competes with VEGF for binding to VEGFR2 and thus attenuates VEGFR2 signaling was already observed by Zhang et al. and Yang et al. [57, 58]. We here, however, observed that the combination of PEDF with VEGF generates new signaling events that are not produced by PEDF or VEGF alone. Thus, while sFlt1 as a classical capture molecule can block VEGF effects, PEDF alters VEGF signaling and can produce additional effects.

Reduced ERK1/2 phosphorylation by VEGF in the presence of PEDF in fact parallels our findings that combination of VEGF and PEDF reduced 2D network formation and proliferation of fpEC and attenuated vascular growth in the chorioallantoic membrane. However, increased FAK phosphorylation at tyrosine 397 in the presence of VEGF and PEDF is difficult to interpret, since FAK phosphorylation and, thus, FAK activity promote cellular movement and proliferation [62, 63]. Thus, in a situation of reduced angiogenesis and proliferation caused by the combination of VEGF and PEDF, increased FAK phosphorylation seems counterintuitive. However, we here determined the effect of PEDF and VEGF on immediate signaling events, investigated only one of several FAK phosphorylation sites and did not analyze downstream effects. Hence, we observed that combination of PEDF and VEGF produces distinct signaling events than both factors alone, but we cannot yet conclude that this combination ultimately stimulates FAK-mediated cellular processes.

More PEDF was expressed in trophoblasts from late pregnancy, whereas sFlt1 secretion was stronger by trophoblasts from early pregnancy. Accordingly, sFlt1 appears to be the predominant regulator of feto-placental angiogenesis in early pregnancy, while PEDF-mediated anti-angiogenic effects prevail in late pregnancy.

The effect of PEDF to limit feto-placental angiogenesis may have implications for pregnancy pathologies that are related to altered feto-placental angiogenesis. Indeed, PEDF expression was decreased in total placental tissue after unexpected stillbirth, a condition associated with vasculopathy and increased angiogenesis [64]. Since we identified trophoblast as the major site of placental PEDF production, this finding provides indirect evidence that trophoblast-derived PEDF is secreted toward the feto-placental endothelium to regulate angiogenesis.

Strength of the study is the use of isolated human primary cells. However, this also entails its limitation. Trophoblast CM from early and late pregnancy were applied on feto-placental endothelial cells from late pregnancy, because methods for the isolation of feto-placental endothelial cells from early pregnancy with high yield and purity have not been established so far. We acknowledge that testing the effect of early pregnancy trophoblast CM on feto-placental endothelial cells also from early pregnancy would have been more appropriate.

To assess the effect of CM and PEDF on angiogenesis, we employed a Matrigel 2D network formation assay. In this highly reproducible assay, cells develop two-dimensional, cord-like structures, which, however, do not necessarily reflect the complex formation of vascular networks in vivo [65]. In order to determine the effect of CM and PEDF on vascular development in vivo, we employed the CAM assay, which allows monitoring of vessel growth in response to pro- and anti-angiogenic factors in real time [65].

In conclusion, trophoblasts, particularly in late pregnancy, secrete PEDF in order to restrict growth and expansion of the feto-placental endothelium. The trophoblast forms the placental epithelium, which is in contact with the maternal circulation, in contrast to the feto-placental endothelium, which has no direct contact with maternal blood. The important implication of our findings is that the maternal environment may affect the feto-placental endothelium through modulating the trophoblast–endothelial paracrine network.

## Electronic supplementary material

Below is the link to the electronic supplementary material.

Supplementary material 1 (PPTX 98 kb)
