# Supplementary material for: Pigment epithelium-derived factor (PEDF): a novel trophoblast-derived factor limiting feto-placental angiogenesis in late pregnancy
Source: Angiogenesis. 2016 Jun 8;19:373–88. doi: 10.1007/s10456-016-9513-x (PMC4930480; doi:10.1007/s10456-016-9513-x)
Supplement: Supplementary file 1 — Supplementary material 1 (PPTX 98 kb) [file 10456_2016_9513_MOESM1_ESM.pptx]

## Slide 1
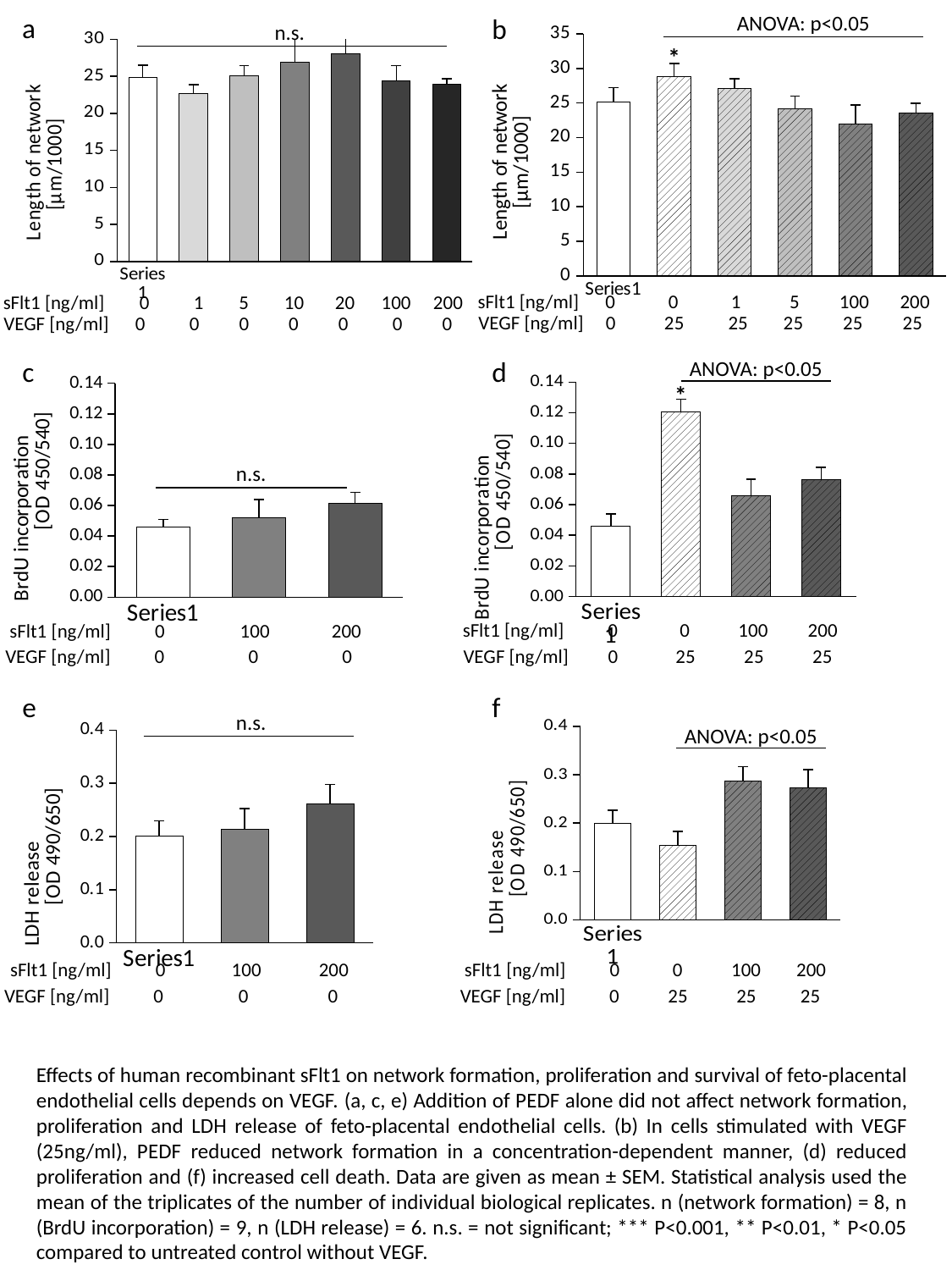

### Chart
| Category | |
|---|---|
| | 24.875 |
| | 22.6875 |
| | 25.1 |
| | 26.9 |
| | 28.0 |
| | 24.43 |
| | 23.914285714285715 |
### Chart
| Category | |
|---|---|
| | 25.18 |
| | 28.78 |
| | 27.02857142857143 |
| | 24.12857142857143 |
| | 21.9375 |
| | 23.5625 |ANOVA: p<0.05
a
b
n.s.
*
sFlt1 [ng/ml] 0 0 1 5 100 200
sFlt1 [ng/ml] 0 1 5 10 20 100 200
VEGF [ng/ml] 0 25 25 25 25 25
VEGF [ng/ml] 0 0 0 0 0 0 0
c
### Chart
| Category | |
|---|---|
| | 0.046 |
| | 0.12065320542756518 |
| | 0.06578728424805448 |
| | 0.07656137011042832 |d
ANOVA: p<0.05
### Chart
| Category | |
|---|---|
| | 0.046 |
| | 0.05193454144615559 |
| | 0.06140049651804977 |*
n.s.
sFlt1 [ng/ml] 0 0 100 200
sFlt1 [ng/ml] 0 100 200
VEGF [ng/ml] 0 25 25 25
VEGF [ng/ml] 0 0 0
e
f
### Chart
| Category | |
|---|---|
| | 0.199999992 |
| | 0.15442440666666668 |
| | 0.28708816000000004 |
| | 0.272942706 |
### Chart
| Category | |
|---|---|
| | 0.19999998571428573 |
| | 0.21277801428571436 |
| | 0.2613927914285714 |n.s.
ANOVA: p<0.05
sFlt1 [ng/ml] 0 100 200
sFlt1 [ng/ml] 0 0 100 200
VEGF [ng/ml] 0 0 0
VEGF [ng/ml] 0 25 25 25
Effects of human recombinant sFlt1 on network formation, proliferation and survival of feto-placental endothelial cells depends on VEGF. (a, c, e) Addition of PEDF alone did not affect network formation, proliferation and LDH release of feto-placental endothelial cells. (b) In cells stimulated with VEGF (25ng/ml), PEDF reduced network formation in a concentration-dependent manner, (d) reduced proliferation and (f) increased cell death. Data are given as mean ± SEM. Statistical analysis used the mean of the triplicates of the number of individual biological replicates. n (network formation) = 8, n (BrdU incorporation) = 9, n (LDH release) = 6. n.s. = not significant; *** P<0.001, ** P<0.01, * P<0.05 compared to untreated control without VEGF.
